# Dynamics of Occupational Well-Being and Turnover Intention in Long-Term Care: A 12-Month Longitudinal Study of Nursing Assistants

**DOI:** 10.3390/healthcare14152316

**Published:** 2026-07-31

**Authors:** Diana Carbone, Miriam Tonetto, Francesco Marcatto

**Affiliations:** Department of Life Sciences, University of Trieste, Via Weiss 2, 34124 Trieste, Italy; diana.carbone@units.it (D.C.); miriam.tonetto@studenti.units.it (M.T.)

**Keywords:** turnover intention, job demands–resources model, emotional exhaustion, job satisfaction, longitudinal design, long-term care

## Abstract

**Highlights:**

**What are the main findings?**

- Turnover intention was associated with both stable between-person differences and within-person changes across measurement occasions.
- Emotional exhaustion, job satisfaction, and social support emerged as complementary strain and motivational factors associated with turnover intention.

**What are the implications of the main findings?**

- A longitudinal multilevel approach helps disentangle stable individual differences from changes occurring within individuals over time.
- Effective retention strategies should address both structural working conditions and the motivational and vocational dimensions of healthcare work.

**Abstract:**

**Background/Objectives**: High turnover intention represents a critical challenge in the long-term care sector, affecting both workforce stability and quality of care. Identifying the psychosocial factors associated with turnover intention and understanding how they operate over time are essential for developing effective retention strategies. This study examined both between-person and within-person associations between psychosocial variables and turnover intention among nursing assistants, focusing on perceived occupational stress, emotional exhaustion, job satisfaction, and social resources. **Methods**: A three-wave longitudinal study was conducted among 52 nursing assistants from a single Italian long-term care facility over a 12-month period. Linear mixed-effects models were used to distinguish within-person (time-varying) and between-person (stable) associations between psychosocial factors and turnover intention. **Results**: At the within-person level, increases in perceived occupational stress, emotional exhaustion, and job satisfaction were associated with changes in turnover intention across measurement occasions. At the between-person level, higher average emotional exhaustion and lower average job satisfaction were associated with higher turnover intention, while social support showed a smaller negative association. **Conclusions**: These findings suggest that turnover intention is associated with both stable between-person differences and changes in psychosocial work experiences over time. More broadly, the study highlights the value of adopting a longitudinal multilevel perspective to simultaneously examine strain indicators, job attitudes, and social resources in understanding turnover intention among nursing assistants working in long-term care.

## 1. Introduction

Population aging represents one of the most significant demographic transformations of the 21st century. Globally, people are living longer, and by 2030, one in six individuals will be aged 60 or older, with the total number of older adults expected to increase substantially in the coming decades [1]. While this shift reflects important advances in health and living conditions, it also poses major challenges for health and social care systems, which must adapt to increasingly complex and long-term care needs through integrated, person-centered models of care [2]. Within this framework, ensuring the sustainability and quality of long-term care systems has become a key global priority, also reflected in the United Nations Agenda 2030 for Sustainable Development [3].

In Italy, nursing assistants working in long-term care facilities play a crucial role in supporting older adults’ daily functioning and overall well-being [4]. Their work combines direct care activities with continuous interaction with residents, families, and other healthcare professionals, requiring both technical competence and strong interpersonal skills. Consequently, nursing assistants are frequently exposed to emotionally demanding situations, and previous research has highlighted how care work in long-term care facilities is characterized by high physical and emotional demands, complex relational dynamics, and intense work rhythms [5]. When such demands are not adequately balanced by organizational support and sufficient resources, such as fair working conditions, recognition, and a functional work environment, workers may experience increased strain. Over time, this imbalance can diminish well-being and heighten turnover intention, driving workforce instability in long-term care settings, as consistently reported in the aged care literature [6,7]. In this context, it is crucial to distinguish between occupational turnover (i.e., leaving the nursing care profession altogether) and organizational turnover (i.e., leaving the current employing facility) [8]. In the long-term care sector, occupational attrition is particularly prominent, with rates often exceeding organizational turnover as care workers leave the profession entirely due to chronic strain and systemic burden [9]. Nevertheless, organizational-level retention remains a critical challenge: in some Italian regions, the annual turnover rate for nursing assistants in long-term care facilities exceeds 20% [10]. This percentage surpasses the threshold generally considered critical for organizational stability [11]. Moreover, high turnover rates are particularly concerning in long-term care settings, as they can negatively affect residents’ well-being. Previous research has shown that frequent staff turnover is associated with disruptions in continuity of care, increased reliance on inexperienced temporary workers, and reduced staff commitment, ultimately leading to adverse physical and emotional outcomes for residents [12]. To address these challenges, the present study explicitly operationalizes turnover intention as organizational turnover intention, specifically, a nursing assistant’s intention to leave their current employing organization, thereby providing targeted insights for organizational-level management and retention strategies.

### 1.1. Theoretical Framework

The Job Demands–Resources (JD-R) model [13,14,15] provides a comprehensive framework for understanding the psychological and work-related mechanisms underlying turnover intention among nursing assistants. According to this model, job characteristics can be broadly categorized into job demands and job resources. Job demands require sustained physical or psychological effort and are therefore associated with physiological and psychological costs, potentially leading to strain and health impairment. In contrast, job resources are instrumental in achieving work goals, fostering motivation, and buffering the negative effects of job demands.

Within this framework, two complementary processes have been identified. The health impairment process suggests that prolonged exposure to high job demands may lead to exhaustion and burnout, ultimately increasing employees’ intention to leave. Conversely, the motivational process posits that job resources enhance work engagement, strengthen organizational attachment, and reduce turnover intention.

In long-term care settings, nursing assistants are exposed to multiple and often interacting job demands. The work environment plays a key role, as factors such as relationships with supervisors, organizational culture, interpersonal dynamics, and organizational communication have been associated with reduced professional effectiveness and higher levels of burnout [16]. In addition to these contextual factors, occupational stress represents a central psychosocial risk factor in long-term care [17] and has been consistently linked to greater emotional exhaustion [18], a core component of burnout [19]. These findings highlight how the accumulation of demands may contribute to the health impairment process, increasing the risk of strain and, ultimately, turnover intention.

Within the motivational process, different psychosocial factors have been associated with turnover intention. These include job attitudes, motivational states, and contextual resources. Job satisfaction, although more appropriately conceptualized as a work-related attitude than as a job resource, is influenced by organizational conditions [20] and has consistently been found to be negatively associated with turnover intention [4,21]. Similarly, work engagement represents a positive motivational state and has been linked to both higher quality of care delivery [22] and lower turnover intention [4]. Another important contextual resource is social support, particularly from colleagues and supervisors. Social support has been associated with lower emotional exhaustion and higher levels of personal accomplishment [18]. It has also been shown to buffer the relationship between occupational stress and turnover intention among healthcare workers [23], highlighting the importance of supportive relationships in emotionally demanding care settings [24]. While past research has heavily focused on managerial support, recent literature highlights the need to systematically investigate coworker support, as it represents an essential proximal resource directly tied to healthcare workers’ motivation and retention [25].

Overall, the JD-R model provides a useful framework for understanding how health impairment and motivational processes are associated with turnover intention among nursing assistants in long-term care. It also highlights the importance of adopting a holistic view of occupational well-being in healthcare settings.

To fully capture how psychosocial working conditions shape turnover intention, it is essential to establish an explicit theoretical alignment between the dual pathways of the JD-R model, the health impairment process and the motivational process [13,14,15], and a two-level analytical framework [26,27]. At the within-person level, this alignment accounts for short-term dynamic processes occurring within individuals over time [26,28]. Specifically, short-term fluctuations in job demands trigger transient elevations in perceived stress and emotional exhaustion via the health impairment pathway, whereas short-term increases in job resources foster immediate improvements in job satisfaction through the motivational pathway [26,29]. Together, these transient, state-like experiences drive within-person fluctuations in turnover intention across time. At the between-person level, this framework reflects long-term cumulative effects [27,29]. Individuals with chronic exposure to high job demands develop persistent, trait-like emotional exhaustion (chronic strain), whereas individuals with sustained access to abundant job resources maintain consistently high baseline job satisfaction and social support (trait-like motivation) [13,15]. These stable, between-person differences account for individual baseline variability in turnover intention.

Integrating the dual processes of the JD-R model within a two-level multilevel framework thus provides a more refined theoretical foundation, clarifying how both short-term situational experiences and stable, chronic work conditions independently contribute to employee retention in long-term care settings.

### 1.2. Well-Being as a Dynamic Process

Importantly, the JD-R framework conceptualizes occupational well-being as a dynamic process. Employees’ levels of stress and motivation constantly evolve in response to changing working conditions. Consequently, their relationships with organizational outcomes may also shift over time.

A longitudinal approach is thus particularly well suited to capturing these temporal dynamics. While between-person differences provide important information about how employees differ from one another, they do not fully account for how individuals’ experiences change over time [26]. In contrast, examining within-person variations allows for a more comprehensive understanding of how fluctuations in working conditions are associated with changes in well-being and work-related outcomes [28].

In this context, multilevel modeling offers a flexible analytical framework for longitudinal data, as it enables the simultaneous examination of within-person changes over time and between-person differences [27]. This approach is particularly valuable in occupational health research, as it allows researchers to move beyond static comparisons and investigate how changes in key variables (such as occupational stress) are associated with changes in outcomes such as turnover intention.

Despite the relevance of these dynamic processes, relatively few studies have adopted a longitudinal approach to examine turnover intention among nursing assistants in long-term care settings [30,31]. Moreover, existing studies have typically relied on two-wave designs. Although such designs are useful, multiwave studies with repeated measurements across three or more time points provide a more robust framework for capturing complex and evolving processes in occupational health psychology [28,32].

### 1.3. Study Objectives and Hypotheses

Previous studies have consistently identified job demands and job resources as key correlates of turnover intention among nursing assistants. However, most evidence comes from cross-sectional studies or longitudinal designs that do not distinguish stable between-person differences from within-person changes over time. As a result, it remains unclear to what extent different psychosocial factors contribute to turnover intention through between-person differences, dynamic within-person changes, or both. Addressing this gap is essential both for refining theoretical models of occupational well-being and for informing interventions aimed at reducing turnover in long-term care settings. The present study aims to examine both stable (between-person) and dynamic (within-person) predictors of turnover intention among nursing assistants working in long-term care facilities over a 12-month period, using a three-wave longitudinal multilevel design.

Two hypotheses were put forward:

**H1.**  *Within-person changes in the psychosocial variables under study will be associated with corresponding changes in turnover intention.*

Specifically, increases in occupational stress and emotional exhaustion are expected to be associated with higher turnover intention, whereas increases in job satisfaction and perceived social support are expected to be associated with lower turnover intention.

**H2.**  *Stable between-person differences in the psychosocial variables examined in the present study will be associated with differences in turnover intention.*

Specifically, individuals reporting higher levels of occupational stress and emotional exhaustion are expected to report higher turnover intention, whereas those reporting higher levels of job satisfaction and perceived social support are expected to report lower turnover intention.

## 2. Materials and Methods

### 2.1. Participants and Procedure

This study employed a longitudinal design and was approved by the Ethics Committee of the University of Trieste (Minutes No. 130, dated 27 April 2023). Participants were nursing assistants working in a long-term care facility for older adults comprising multiple residential units. They were recruited in May 2023 during an internal training course, where they received the baseline questionnaire (T0). The second wave of data collection took place in November 2023 (T1), and the third in May 2024 (T2). Eligible participants were selected by the organization from nursing assistants who were actively employed in the residential care units during the recruitment period and had not previously participated in similar training workshops. No minimum organizational tenure was required, and both permanent and fixed-term employees were eligible. Although recruitment took place in conjunction with a mandatory training program, participation in the research component was entirely voluntary.

At each measurement occasion, questionnaires were administered in a paper-and-pencil format during the scheduled training sessions. The first page of each questionnaire included written informed consent, specifying that participation was voluntary, that participants could withdraw at any time without providing a reason, and that all responses were anonymous, with only aggregated results shared with the organization. Participants were required to check “I consent” to proceed. To match responses across waves while preserving anonymity, participants were asked to generate a personal code consisting of two letters and two numbers to be used across all questionnaires.

**Figure 1:**
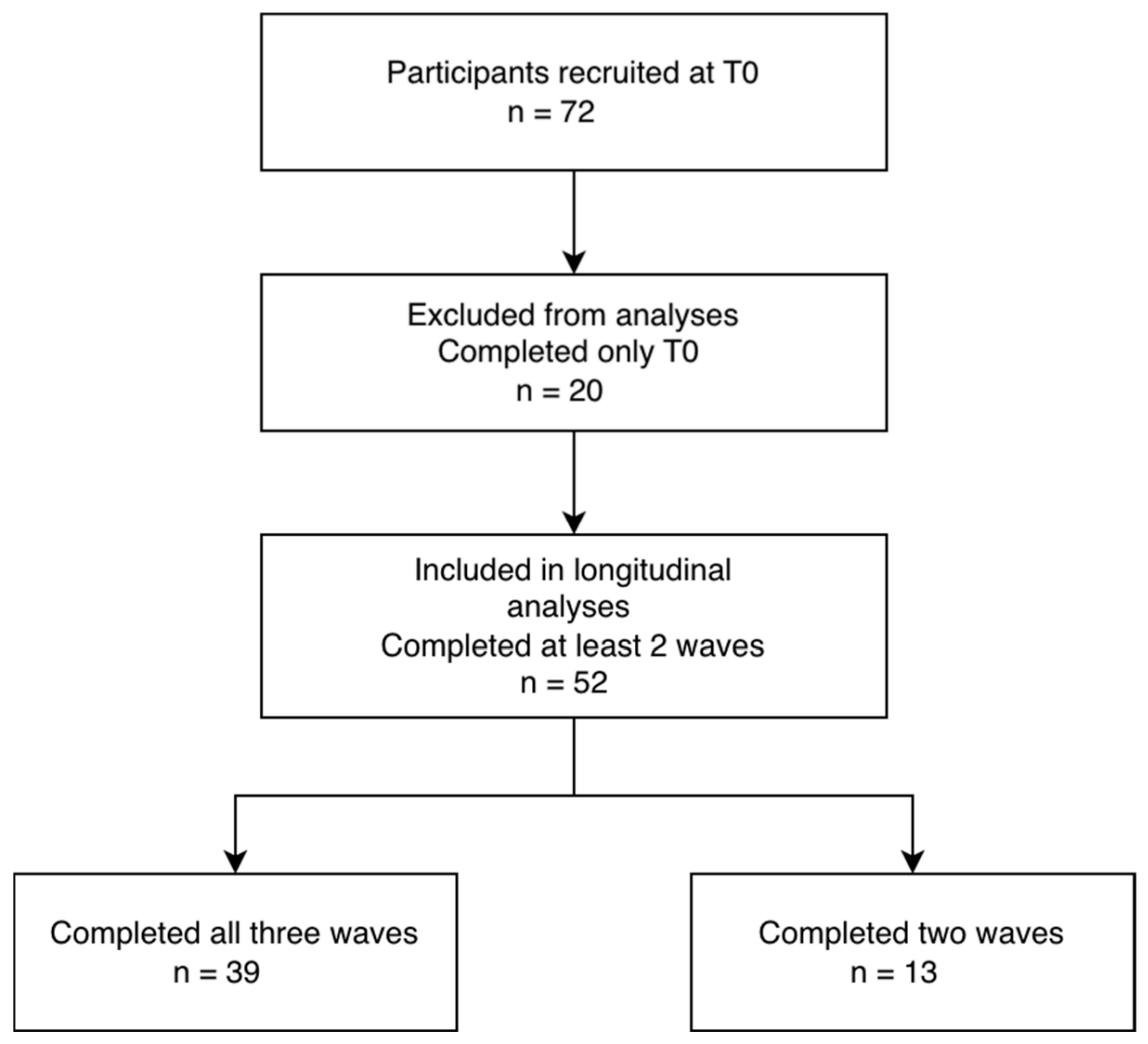
Flow diagram of participant inclusion in the longitudinal analyses.

A total of 72 participants were recruited at T0. Participants who completed only the baseline assessment (*n* = 20) were excluded from the longitudinal analyses because the decomposition of predictors into within-person (person-mean-centered) and between-person components requires repeated observations for each participant. With only a single measurement occasion, person-specific deviations from the individual mean (i.e., within-person variability) cannot be estimated. Furthermore, using a single observation as a proxy for a participant’s stable average (between-person component) would yield a less reliable estimate of stable individual differences, as it reflects a single measurement occasion rather than the participant’s average across repeated observations. The final analytical sample thus comprised 52 participants who completed at least two measurement waves. A flow diagram of participant inclusion is presented in Figure 1.

Given that the study was embedded within a training program, attrition was partly due to organizational factors. Specifically, several participants held fixed-term contracts that expired during the summer period, preventing them from completing the training and, consequently, the subsequent waves of data collection. This likely contributed to the observed attrition. To assess potential attrition bias, baseline demographic characteristics and study variables were compared between participants retained in the analytical sample (≥2 waves) and those excluded because they completed only one assessment. The results of these comparisons are reported in the Results section (see also Supplementary Table S1).

### 2.2. Measures

**Work-related stress**. The Perceived Occupational Stress scale (POS) [33] was employed to measure the level of stress experienced at work. A 5-point Likert scale was used to rate the four items (from 1, “strongly disagree” to 5, “strongly agree”). The total score was calculated as the average of item responses, with higher scores indicating a greater level of occupational stress. Cronbach’s alpha was 0.79 at T0 and 0.78 at T1 and T2.

**Turnover intention**. The Turnover Intention measure (TI) [34] is a three-item scale assessing turnover intention intensity, based on Mobley and colleagues’ definition [35,36], with responses rated on a 5-point Likert scale (from 1, “strongly disagree” to 5, “strongly agree”). The total score was calculated as the average of item responses, with higher scores indicating stronger turnover intention. Cronbach’s alpha was 0.93 at T0, 0.89 at T1, and 0.71 at T2.

**Work engagement**. The short version of the Utrecht Work Engagement Scale (UWES) [37] was used to assess work engagement. This three-item scale was rated on a 7-point Likert frequency scale (from 1, “never” to 7, “always”). Individual scores were averaged to calculate the work engagement total score, with higher scores indicating stronger engagement. Cronbach’s alpha was 0.63 at T0, 0.64 at T1, and 0.70 at T2.

**Burnout**. The Emotional Exhaustion subscale of the Maslach Burnout Inventory (MBI) [38] questionnaire was used to assess the core dimension of perceived burnout. This 9-item scale was rated on a 7-point Likert frequency scale (from 1, “never” to 7, “always”). Scores were averaged, with higher scores indicating higher emotional exhaustion. Cronbach’s alpha was 0.90 at T0, 0.92 at T1, and 0.86 at T2.

**Occupational stressors and resources**. The Health and Safety Executive Management Standards Indicator Tool (HSE-MS IT) [39] is a 35-item questionnaire aimed at evaluating employees’ exposure to a set of occupational stressors, according to the Management Standards developed in the United Kingdom by the Health and Safety Executive. The HSE-MS IT evaluates seven intercorrelated scales: Demands (8 items, α = 0.75 at T0, α = 0.60 at T1, and α = 0.60 at T2), Control (6 items, α = 0.75 at T0, α = 0.73 at T1, and α = 0.78 at T2), Managers’ support (5 items, α = 0.86 at T0, α = 0.89 at T1, and α = 0.90 at T2), Peer support (4 items, α = 0.83 at T0, α = 0.75 at T1, and α = 0.80 at T2), Relationships (4 items, α = 0.66 at T0, α = 0.66 at T1, and α = 0.69 at T2), Role (5 items, α = 0.82 at T0, α = 0.73 at T1, and α = 0.83 at T2), and Change (3 items, α = 0.42 at T0, α = 0.64 at T1, and α = 0.53 at T2). Items were evaluated on a 5-point Likert frequency scale (from 1, “never” to 5, “always”). Higher scores in the HSE-MS IT scales indicate lower exposure to stressors. Due to its low reliability, the Change subscale was not included in the analyses. This subscale is known to be the weakest dimension of the Italian version of the HSE-MS IT [40,41], a limitation also reported in previous research [42].

**Job satisfaction**. The Satisfaction with Work Scale (SwWS) [43] was used to measure general job satisfaction, modeled after the popular Satisfaction with Life Scale developed by Diener and colleagues [44]. The scale comprised five items rated on a 6-point Likert scale (from 1, “strongly disagree” to 6, “strongly agree”). Individual scores were averaged to calculate the job satisfaction total score, with higher scores indicating higher satisfaction. Cronbach’s alpha was 0.86 at T0, 0.77 at T1, and 0.80 at T2.

**Demographic and employment characteristics**. Information on participants’ gender, age, organizational tenure, and contract type was collected at baseline. Gender was coded as female (0) or male (1). Age was assessed using five categories (18–29, 30–39, 40–49, 50–59, and ≥60 years). Organizational tenure was recorded in six categories (0–1, 2–5, 6–10, 10–20, 20–30, and >30 years). Contract type was coded as permanent (1) or fixed-term (0).

Overall, most measures showed acceptable internal consistency across measurement occasions. Lower reliability was observed for work engagement and for some HSE-MS IT subscales, particularly Change. These psychometric limitations were fully taken into account when interpreting the study findings.

### 2.3. Model Building Strategy

To examine the association between psychosocial factors and turnover intention over time, a series of linear mixed-effects models was estimated, with repeated measurements (Level 1) nested within individuals (Level 2). Degrees of freedom and *p*-values for fixed effects were estimated using Satterthwaite’s approximation to account for the unbalanced structure of the data and the relatively small Level-2 sample size. A null model including only a random intercept for participants was first estimated to assess the proportion of variance attributable to between-person differences. The intraclass correlation coefficient (ICC = 0.62) indicated that a substantial portion of the variance in turnover intention was explained by stable between-person differences, supporting the use of a multilevel approach. Time (occasion) was then included as a fixed effect to control for potential linear trends across measurement waves. However, its effect was not statistically significant and did not improve model fit. Although the inclusion of a random slope for time was explored and resulted in a marginal improvement in model fit, the fixed effect of time remained non-significant, and the added complexity was not central to the research questions. Therefore, a more parsimonious random-intercept model structure was retained.

Given the relatively small sample size and the substantial intercorrelations among predictors, model building followed a parsimonious and theory-driven approach. Predictors were decomposed into within-person (person-mean-centered) and between-person (person-mean) components. For the selection of between-person predictors, priority was given to theoretically central variables reflecting distinct aspects of the stress–motivation framework, while minimizing redundancy among predictors. Specifically, mean emotional exhaustion was retained as a key indicator of chronic occupational strain because it represents the core manifestation of the health impairment process in the Job Demands–Resources framework and has consistently been identified as one of the most proximal psychological predictors of turnover intention [29,45]. Mean job satisfaction was retained as the motivational indicator because it is theoretically more proximal to withdrawal cognitions and turnover intention than work engagement and has consistently shown strong associations with voluntary turnover in previous research [46,47]. Among the HSE-MS IT dimensions, peer support was retained because it captures the immediate social resources available in everyday work interactions and represents a contextual resource conceptually distinct from chronic strain and motivational evaluations [48]. No data-driven stepwise selection procedure was applied to between-person predictors. Instead, predictor selection was based primarily on theoretical considerations, with preliminary correlation analyses used solely to verify that the retained variables showed consistent associations with turnover intention while minimizing redundancy among conceptually related constructs. This strategy was adopted a priori to preserve model interpretability and reduce the risk of overfitting given the limited number of Level-2 observations. For within-person predictors, separate models were initially estimated for (a) occupational stress and strain indicators (perceived occupational stress and emotional exhaustion), (b) HSE-MS IT dimensions, and (c) motivational and attitudinal variables (job satisfaction and work engagement). Only predictors showing consistent associations with turnover intention were retained in the final model. To examine the robustness of the final model, additional demographic and employment-related variables (gender, age, and contract type) were subsequently entered as Level-2 covariates. Because these variables represent relatively stable demographic and employment characteristics rather than psychological constructs derived from the JD-R framework, they were included only as supplementary robustness controls to evaluate whether the associations between the psychological predictors and turnover intention remained stable after accounting for differences in sample composition. Finally, the robustness of the theoretically motivated Level-2 specification was evaluated by estimating alternative models using conceptually related indicators of the same theoretical processes (e.g., perceived occupational stress instead of emotional exhaustion, work engagement instead of job satisfaction, and managerial support instead of peer support). The number of observations varied slightly across models because mixed-effects models were estimated using all available observations with complete data for the variables included in each analysis. All analyses were conducted using Jamovi software (Version 2.6.25).

## 3. Results

### 3.1. Sample Characteristics

Figure 1 illustrates participant inclusion and retention throughout the study. The final analytical sample comprised 52 participants, of whom 39 completed all three measurement waves and 13 completed two. The distributions of age, gender, organizational tenure, and contract type are summarized in Table 1. Overall, demographic characteristics and organizational tenure of the analytical sample remained relatively stable across measurement waves. In contrast, the proportion of participants with permanent contracts increased over time, reflecting both the attrition of fixed-term employees and, for a small number of participants, transitions to permanent employment during the follow-up period.

### 3.2. Attrition Analysis and Descriptive Statistics

Attrition analyses indicated that participants retained in the analytical sample did not differ significantly from excluded participants in terms of baseline demographic characteristics (gender, age, and organizational tenure) or most baseline psychological variables, including perceived occupational stress, emotional exhaustion, turnover intention, work engagement, job satisfaction, managerial support, and HSE demands (all *p*-values > 0.05). The only statistically significant difference was observed for peer support, with retained participants reporting higher baseline peer support than excluded participants (*p* = 0.022, *d* = 0.64). Participants with permanent contracts were more likely to be retained in the study, although this difference did not reach statistical significance (*p* = 0.067). Full attrition analyses are reported in Supplementary Table S1.

Descriptive statistics for all study variables across the three measurement occasions are reported in Table 2. Mean levels of perceived occupational stress and emotional exhaustion decreased slightly over time, whereas job satisfaction and work engagement remained relatively stable. Turnover intention decreased slightly from T0 to T1 and remained relatively stable at T2. Regarding the HSE-MS IT dimensions, demands showed a slight increase in mean values across occasions, reflecting a lower perceived exposure to demands over time, whereas control and managerial support displayed modest fluctuations. Peer support and relationships increased from T0 to T1 before stabilizing at T2, while role clarity remained consistently high throughout the observation period.

Zero-order correlations among the study variables at baseline (T0) are presented in Table 3. As expected, turnover intention was strongly and positively correlated with emotional exhaustion (*r* = 0.74, *p* < 0.001) and perceived occupational stress (*r* = 0.52, *p* < 0.001), while displaying strong negative correlations with job satisfaction (*r* = −0.66, *p* < 0.001), work engagement (*r* = −0.50, *p* < 0.001), and structural resources such as demands (*r* = −0.51, *p* < 0.001) and peer support (*r* = −0.47, *p* < 0.001).

Because all study variables were assessed using self-report measures, Harman’s single-factor test was conducted as an additional diagnostic analysis. The first unrotated factor accounted for 29.8% of the total variance, suggesting that common method variance was unlikely to have substantially influenced the observed associations.

### 3.3. Preliminary Within-Person Models

In the model examining within-person occupational stress and strain variables, both perceived occupational stress (*b* = 0.43, *SE* = 0.14, *p* = 0.003) and emotional exhaustion (*b* = 0.25, *SE* = 0.10, *p* = 0.014) were positively associated with turnover intention. This indicates that increases in these variables relative to an individual’s own mean were associated with higher turnover intention. Collinearity diagnostics revealed no concerns regarding multicollinearity (VIFs = 1.29), suggesting that perceived stress and emotional exhaustion captured complementary variance in turnover intention. Overall, the fixed effects explained 7.2% of the total variance (marginal *R*^2^ = 0.072, *p* < 0.001; conditional *R*^2^ = 0.720).

In contrast, none of the within-person HSE-MS IT dimensions were significantly associated with turnover intention when entered simultaneously into the model (all *p*-values > 0.10). The fixed effects explained a small and non-significant portion of the variance (marginal *R*^2^ = 0.051, *p* = 0.065). Collinearity diagnostics showed no multicollinearity concerns among HSE predictors (all VIFs < 1.6), suggesting that the lack of significant effects was not due to statistical overlap between variables. These findings suggest that within-person changes over time in HSE-assessed psychosocial working conditions were not significantly associated with turnover intention in the present sample. However, given the relatively low reliability of some HSE-MS IT subscales and the limited sample size, these null findings should be interpreted cautiously.

In the model evaluating motivational and attitudinal variables, job satisfaction was negatively associated with turnover intention, such that higher-than-usual satisfaction was associated with lower turnover intention (*b* = −0.48, *SE* = 0.11, *p* < 0.001). In contrast, work engagement was not significantly related to turnover intention (*p* = 0.860). The fixed effects explained 5.4% of the variance (marginal *R*^2^ = 0.054, *p* < 0.001). Although within-person changes in job satisfaction and work engagement were moderately correlated (*r* = 0.30), only job satisfaction showed a significant within-person association with turnover intention in the present sample.

The full results of these preliminary models are reported in Tables S2–S4 of the Supplementary Material.

### 3.4. Final Within-Person Model

The predictors that showed significant associations in the preliminary analyses were entered into a final within-person model. All three predictors remained significant when entered simultaneously. Specifically, increases in perceived occupational stress (*b* = 0.35, *SE* = 0.15, *p* = 0.017) and emotional exhaustion (*b* = 0.20, *SE* = 0.10, *p* = 0.046) were associated with higher turnover intention, whereas increases in job satisfaction were associated with lower turnover intention (*b* = −0.27, *SE* = 0.10, *p* = 0.013). Time (occasion) did not show a significant effect. Fixed effects explained 8.5% of the variance (marginal *R*^2^ = 0.085, *p* < 0.001), while the conditional *R*^2^ was 0.735.

The results of the final within-person model are summarized in Table 4.

### 3.5. Final Model Including Within- and Between-Person Predictors

Consistent with the model-building strategy described in the Materials and Methods section, emotional exhaustion, job satisfaction, and peer support were included as the between-person predictors. Preliminary between-person correlations supported this selection, as these variables also showed the strongest associations with turnover intention within their respective domains (*r* = 0.74, *r* = −0.66, and *r* = −0.47, respectively; see Table 3). At the within-person level, perceived occupational stress (*b* = 0.34, *SE* = 0.15, *p* = 0.023), emotional exhaustion (*b* = 0.26, *SE* = 0.10, *p* = 0.014), and job satisfaction (*b* = −0.25, *SE* = 0.10, *p* = 0.018) remained significant predictors of turnover intention. At the between-person level, higher mean emotional exhaustion (*b* = 0.40, *SE* = 0.09, *p* < 0.001) and lower mean job satisfaction (*b* = −0.32, *SE* = 0.09, *p* < 0.001) were associated with higher turnover intention. Additionally, peer support showed a small but significant negative association (*b* = −0.31, *SE* = 0.16, *p* = 0.049).

The normality of residuals was assessed through visual inspection of the Q–Q plot. The distribution appeared approximately normal, with only minor deviations in the tails, supporting the adequacy of model assumptions (see Figure S1 in the Supplementary Materials). The final model explained a substantial proportion of variance (marginal *R*^2^ = 0.575; conditional *R*^2^ = 0.707, *p* < 0.001). In addition, the intraclass correlation coefficient was markedly reduced compared to the null model (ICC = 0.31), indicating that both within- and between-person predictors accounted for a large share of the variability in turnover intention. The results of the final model are summarized in Table 5.

Additional analyses were conducted to test the buffering hypothesis proposed by the Job Demands–Resources framework by examining interactions between indicators of the health impairment process (emotional exhaustion and perceived occupational stress) and selected variables related to the motivational process (peer support and job satisfaction). None of the interaction terms reached conventional levels of statistical significance (see Supplementary Table S5). Nevertheless, among the tested interactions, the within-person emotional exhaustion × between-person job satisfaction term yielded the largest interaction estimate (b = −0.17, 95% CI [−0.34, 0.005], *p* = 0.058). Given the limited sample size, this result should be interpreted cautiously and regarded as preliminary.

### 3.6. Random Slopes

The inclusion of random slopes for within-person predictors was also explored. A random slope for emotional exhaustion resulted in a near-singular fit and did not improve model fit, suggesting minimal variability in the within-person effect across individuals, a pattern likely reinforced by the relatively small sample size. Moreover, the likelihood ratio test showed no significant improvement in model fit. A random slope for perceived occupational stress was also tested. Although the model converged without warnings, the likelihood ratio test again showed no significant improvement in model fit, further indicating limited variability in within-person effects across individuals. Therefore, random slopes were not retained, and the final model included only random intercepts for participants.

### 3.7. Robustness Analysis

To evaluate the robustness of the findings, the final model was re-estimated by simultaneously including gender, age, and baseline contract type as additional Level-2 covariates. Gender and age were not significantly associated with turnover intention (all *p*s > 0.60 and omnibus *p* = 0.970, respectively). In contrast, employees with permanent contracts reported lower turnover intention than those with fixed-term contracts (*b* = −0.53, *SE* = 0.22, *p* = 0.019). Importantly, the inclusion of these covariates did not materially alter the pattern of associations observed for the psychological predictors. The estimated coefficients for within-person perceived occupational stress, emotional exhaustion, and job satisfaction, as well as between-person emotional exhaustion and job satisfaction, remained highly comparable to those obtained in the primary model. The estimated coefficient for between-person peer support remained comparable in magnitude, although its confidence interval included zero after adjustment. Overall, these findings support the robustness of the main results after controlling for demographic and employment-related characteristics. Full results of the robustness analyses are reported in Supplementary Table S6.

Finally, to further evaluate the robustness of the final model specification, alternative Level-2 models were estimated using conceptually related indicators of the same theoretical processes (e.g., perceived occupational stress instead of emotional exhaustion, work engagement instead of job satisfaction, and managerial support instead of peer support). Replacing emotional exhaustion with perceived occupational stress and job satisfaction with work engagement resulted in a poorer-fitting model, as indicated by lower marginal *R*^2^, higher residual between-person variance (ICC), and less favorable information criteria (AIC/BIC). In contrast, replacing peer support with managerial support produced a largely comparable pattern of results, suggesting that the findings were robust to the operationalization of the social resource construct. Taken together, these findings provide additional support for the theoretically motivated specification of the final Level-2 model. Full results of the alternative model specifications are reported in Supplementary Table S7.

## 4. Discussion

The present study examined both stable and dynamic predictors of turnover intention among nursing assistants working in long-term care, using a three-wave longitudinal design. Overall, the findings suggest that turnover intention is shaped by both stable individual differences and changes occurring within individuals over time. At the within-person level, increases in emotional exhaustion and perceived occupational stress were associated with corresponding increases in turnover intention, whereas higher-than-usual levels of job satisfaction were associated with a lower intention to leave. At the between-person level, individuals reporting higher average levels of emotional exhaustion showed greater turnover intention, while higher levels of job satisfaction and peer support were associated with a lower propensity to leave. Importantly, these findings remained robust after controlling for demographic and employment-related characteristics and were largely consistent across alternative model specifications.

The pattern of findings is broadly consistent with the health impairment process proposed by the Job Demands–Resources (JD-R) model [13,15]. Specifically, both temporary increases in perceived occupational stress and emotional exhaustion, as well as higher average levels of emotional exhaustion, were associated with greater turnover intention. This suggests that both transient and more chronic manifestations of occupational strain may contribute to employees’ intention to leave. Conversely, the associations observed for job satisfaction at both levels of analysis are consistent with the motivational process proposed by the JD-R model. They highlight the role of positive work-related experiences in fostering organizational attachment and reducing turnover intention.

Peer support also showed a small but statistically significant association at the between-person level. This suggests that stable differences in the social work environment may play a protective role, even if their effects are less pronounced than those of individual motivational factors. Interestingly, alternative model specifications indicated that the observed protective role of social resources was not specific to peer support alone. Replacing peer support with managerial support produced a largely comparable pattern of results. This could indicate that the beneficial role of social resources does not depend on a single operationalization. However, contrary to expectations derived from the Job Demands–Resources framework, the additional interaction analyses provided only limited evidence for buffering effects. Although none of the tested interactions reached conventional levels of statistical significance, the within-person emotional exhaustion × between-person job satisfaction term showed the largest negative interaction estimate, suggesting a possible buffering pattern. Nevertheless, the evidence remained inconclusive. Given the relatively small sample size, this finding should be interpreted cautiously and requires replication in larger longitudinal samples. Overall, the present findings more consistently support the independent contributions of psychosocial demands and resources than their hypothesized interactive effects.

Beyond supporting the JD-R framework, these findings are consistent with previous research on nursing assistants [4,17,18,23,30,49,50] and extend the existing literature by showing that the associations between psychosocial factors and turnover intention hold not only at a cross-sectional level but also over time, within a longitudinal and multilevel framework.

The main contribution of this study lies in its examination of turnover intention within a longitudinal multilevel framework. Methodologically, the study highlights the value of distinguishing within- and between-person processes when investigating organizational phenomena. The findings indicate that turnover intention is associated not only with stable individual differences but also with changes in employees’ work experiences over time, supporting the view that turnover intention represents a dynamic process rather than a purely stable outcome [51]. Emotional exhaustion and job satisfaction showed associations at both levels of analysis, whereas perceived occupational stress was associated only with changes over time and peer support only with stable individual differences. This pattern highlights the importance of distinguishing between proximal experiences and more stable contextual characteristics when modeling turnover processes. In particular, perceived stress may primarily reflect short-term situational experiences, whereas emotional exhaustion captures both transient increases in strain and more chronic manifestations of occupational strain.

An unexpected finding concerns the lack of a significant association between work engagement and turnover intention, contrary to what would be expected based on previous research [4]. A plausible explanation may lie in the conceptual and empirical overlap between work engagement and job satisfaction. In the current sample, these variables were strongly correlated at the between-person level (*r* = 0.75, *p* < 0.001), suggesting that job satisfaction may have accounted for a substantial portion of the shared variance, thereby reducing the unique contribution of work engagement in the model. Finally, it should be noted that work engagement showed relatively low internal consistency at T0 and T1, which may have attenuated its association with turnover intention. Accordingly, the absence of a significant effect should be interpreted with caution, and conclusions regarding the role of work engagement should remain tentative pending replication in larger longitudinal samples.

Another noteworthy finding concerns the lack of significant within-person effects for the variables assessed through the HSE-MS IT questionnaire. These measures capture psychosocial characteristics of the work environment (such as workload, control, relationships, and support) that may reflect relatively stable features of the work environment rather than experiences that change across measurement occasions. One possible interpretation is that these factors primarily distinguish between individuals or work contexts rather than explaining short-term changes in turnover intention. This view is also consistent with the finding that peer support, one of the HSE-MS IT dimensions, emerged as a significant predictor at the between-person level, but not at the within-person level. However, this explanation remains tentative, given the relatively low internal consistency observed for some HSE-MS IT subscales and the limited temporal resolution of the present three-wave design. These methodological considerations may have reduced the sensitivity to detect within-person associations for some HSE-MS IT dimensions. Despite these limitations, this pattern may indicate that proximal experiences such as stress, emotional exhaustion, and job satisfaction could be more closely associated with turnover intention across measurement occasions. In contrast, broader psychosocial characteristics of the work environment may exert their influence in a more stable and contextual manner. If confirmed in future longitudinal studies, this distinction may help clarify the different roles of experience-based and structural characteristics of the work environment when examining turnover processes [47].

### 4.1. Practical Implications

From a practical standpoint, the findings of this study highlight the necessity of adopting a dynamic approach to prevention, focusing not only on who is at risk (i.e., between-person baseline differences) but also on when risk is elevated (i.e., within-person fluctuations during periods of increased strain). To maximize operational feasibility and retention in long-term care facilities, management strategies should be explicitly structured into two complementary levels: long-term structural strategies (corresponding to between-person effects) and short-term dynamic interventions.

#### 4.1.1. Long-Term Structural Strategies (Between-Person Level)

To address stable, chronic workplace challenges and reduce baseline turnover risk, healthcare administrators should focus on primary prevention through structural organizational improvements:

- Managing Structural Demands and Staffing: Facility management should optimize workload, shift organization, and staffing ratios to reduce chronic sources of strain, as these structural factors are well-documented to negatively affect occupational well-being among healthcare workers [52].
- Improving External and Working Conditions: Enhancing contractual terms, career development opportunities, and overall working conditions is essential, as long-term decisions to remain in an organization are strongly driven by these external structural conditions [53].

#### 4.1.2. Short-Term Dynamic Interventions (Within-Person Level)

In addition to structural policies, management must deploy timely secondary and tertiary interventions to mitigate transient fluctuations in employee strain and turnover intention:

- Periodic Well-Being Monitoring: Organizations should implement brief periodic well-being surveys or regular monitoring systems to promptly detect critical within-person fluctuations in employee strain and intervene before intention to leave escalates.
- Strengthening Adaptive Coping Mechanisms: Facilities should offer targeted training and support aimed at strengthening adaptive coping strategies among nursing assistants [54,55], helping employees effectively manage temporary increases in stress and emotional exhaustion.
- Sustaining Intrinsic Motivation and Job Satisfaction: Because altruistic motivation and intrinsic rewards contribute significantly to job satisfaction even beyond financial compensation [56,57], managers should implement brief team-based reflective sessions, team discussions, or supervision. Reconnecting employees with the core purpose and value of caregiving helps sustain intrinsic motivation and buffers against temporary operational stress.

### 4.2. Limitations and Future Directions

This study has several limitations that should be considered when interpreting the findings. First, regarding the sample and context, the study was conducted within a single long-term care organization, and the analytical sample was determined by the size of the available workforce rather than an a priori recruitment target. The sample size, particularly the number of participants completing all three waves, was relatively small, limiting external validity and generalizability. Contextual factors, such as specific management practices, organizational culture, local labor market dynamics, or unmeasured organizational changes during the 12-month period, may have influenced employees’ experiences. Additionally, although attrition was largely driven by contract expirations rather than systematic dropout, retained participants reported slightly higher baseline peer support. Given that peer support was a significant predictor, this difference represents a potential source of residual selection bias. Future studies involving larger samples across multiple healthcare organizations are recommended. Second, regarding causal inference, the present longitudinal design relied on contemporaneous associations within each measurement wave. Consequently, while it captured within-person fluctuations over time, it cannot establish strict temporal precedence or fully rule out reverse causality between psychosocial factors and turnover intention. To verify the directionality and reciprocal nature of these dynamic relationships, future research should adopt cross-lagged panel designs, such as Random Intercept Cross-Lagged Panel Models. Third, the choice of measurement interval represents a potential constraint. The six-month interval between waves was dictated by operational feasibility and may not align perfectly with the natural, short-term fluctuation cycle of strain and turnover intention. Future studies could employ experience sampling methods or ecological momentary assessment to capture dynamic, within-person processes at a finer temporal scale. Fourth, all variables in this study were assessed via self-report measures, which may introduce common method variance and subjective perceptual bias. Future research should combine self-reports with objective organizational indicators, such as actual turnover behavior, attendance/absenteeism records, and objective workload indicators, to validate subjective perceptions against concrete organizational metrics. Fifth, statistical power constraints restricted the number of predictors that could be included simultaneously in the multilevel models, necessitating a parsimonious modeling strategy. Although robustness analyses confirmed the stability of the findings and the selection strategy was theory-driven, future research with larger samples should test more complex models, including potential multi-level buffering effects. Furthermore, the internal consistency of certain subscales (e.g., work engagement) was lower than standard benchmarks, which may have attenuated some observed associations. Finally, this study focused strictly on organizational turnover intention (leaving the facility). However, occupational turnover (leaving the healthcare profession entirely) represents a critical challenge in long-term care. Future research should extend this two-level framework to compare organizational versus occupational turnover intention, helping administrators distinguish between factors driving facility switching and those leading to complete workforce departure.

## 5. Conclusions

In conclusion, this study highlights the value of integrating strain indicators, job attitudes, and social resources within a longitudinal multilevel framework to better understand turnover intention in long-term care. It suggests that understanding turnover intention requires considering not only stable differences between employees but also dynamic changes in their psychosocial work experiences over time. These findings may inform future research aimed at developing and evaluating organizational strategies to promote workforce stability, including longitudinal monitoring of employee well-being and timely organizational interventions.

## Figures and Tables

**Table 1:** Demographic characteristics of the sample across the three measurement waves.

Characteristic	T0	T1	T2
Age, %			
18–29	38.8%	40%	43.9%
30–39	16.3%	16%	12.2%
40–49	18.4%	18%	22%
50–59	20.4%	20%	19.5%
≥60	6.1%	6%	2.4%
Gender, %			
Female	75.5%	76%	75.6%
Male	24.5%	24%	24.4%
Organizational tenure, %			
0–1	26.5%	22.4%	17.9%
2–5	32.7%	30.6%	35.9%
6–10	8.2%	12.2%	15.4%
10–20	24.5%	26.5%	20.5%
20–30	4.1%	6.1%	7.7%
>30	4.1%	2.0%	2.6%
Employment contract			
Permanent	76.6%	82.2%	91.9%
Fixed-term	23.4%	17.8%	8.1%

**Table 2:** Descriptive statistics for the study variables across the three measurement occasions.

Variable	Mean (SD)
T0 HSE Demands	2.76 (0.64)
T1 HSE Demands	3.04 (0.54)
T2 HSE Demands	3.02 (0.47)
T0 HSE Control	2.48 (0.71)
T1 HSE Control	2.79 (0.77)
T2 HSE Control	2.59 (0.77)
T0 HSE Managerial Support	3.06 (0.95)
T1 HSE Managerial Support	3.17 (1.00)
T2 HSE Managerial Support	3.25 (0.99)
T0 HSE Peer Support	3.55 (0.78)
T1 HSE Peer Support	3.90 (0.65)
T2 HSE Peer Support	3.86 (0.56)
T0 HSE Relationships	3.46 (0.70)
T1 HSE Relationships	3.81 (0.59)
T2 HSE Relationships	3.86 (0.61)
T0 HSE Role	4.39 (0.53)
T1 HSE Role	4.43 (0.51)
T2 HSE Role	4.40 (0.50)
T0 Emotional Exhaustion	3.15 (1.38)
T1 Emotional Exhaustion	2.86 (1.41)
T2 Emotional Exhaustion	2.90 (1.04)
T0 Perceived Occupational Stress	3.08 (1.00)
T1 Perceived Occupational Stress	2.74 (0.97)
T2 Perceived Occupational Stress	2.79 (0.94)
T0 Turnover Intention	2.29 (1.35)
T1 Turnover Intention	1.97 (1.11)
T2 Turnover Intention	2.02 (0.92)
T0 Work Engagement	4.88 (1.36)
T1 Work Engagement	4.87 (1.46)
T2 Work Engagement	4.61 (1.31)
T0 Job Satisfaction	5.11 (1.36)
T1 Job Satisfaction	5.32 (1.10)
T2 Job Satisfaction	5.16 (1.18)

**Table 3:** Zero-order correlations among the study variables at baseline (T0).

Variable	1	2	3	4	5	6	7	8	9	10
1. Turnover intention	—									
2. HSE Control	−0.35 ***	—								
3. HSE Demands	−0.51 ***	0.59 ***	—							
4. HSE Peer Support	−0.47 ***	0.35 ***	0.32 ***	—						
5. HSE Managerial Support	−0.45 ***	0.57 ***	0.57 ***	0.47 ***	—					
6. HSE Role	−0.40 ***	0.16	0.42 ***	0.15	0.26 **	—				
7. HSE Relationships	−0.29 ***	0.08	0.37 ***	0.43 ***	0.21 *	0.34 ***	—			
8. Job Satisfaction	−0.66 ***	0.29 ***	0.56 ***	0.29 ***	0.30 ***	0.48 ***	0.22 *	—		
9. Perceived Occupational Stress	0.52 ***	−0.50 ***	−0.58 ***	−0.34 ***	−0.33 ***	−0.13	−0.36 ***	−0.49 ***	—	
10. Emotional Exhaustion	0.74 ***	−0.44 ***	−0.59 ***	−0.39 ***	−0.39 ***	−0.30 ***	−0.25 **	−0.52 ***	0.78 ***	—
11. Work Engagement	−0.50 ***	0.29 **	0.51 ***	0.34 ***	0.38 ***	0.51 ***	0.18 *	0.76 ***	−0.30 ***	−0.47 ***

Note: * *p* < 0.05, ** *p* < 0.01, *** *p* < 0.001.

**Table 4:** Final within-person model.

	b	SE	95% CI	df	t	*p*
Intercept	2.13	0.14	[1.84; 2.41]	50.8	14.94	<0.001
*Level 1 variables _WP_*						
Occasion	−0.04	0.07	[−0.18; 0.09]	77.2	−0.62	0.536
Perceived occupational stress _WP_	0.35	0.15	[0.06; 0.64]	77.4	2.43	0.017
Emotional exhaustion _WP_	0.20	0.10	[0.004; 0.40]	75.2	2.02	0.046
Job satisfaction _WP_	−0.27	0.10	[−0.47; −0.06]	76	−2.56	0.013

Note: _WP_ = within-person. Number of observations = 129. Number of participants = 52. Random intercept SD = 0.94. Marginal R^2^ = 0.085. Conditional R^2^ = 0.735. Degrees of freedom were estimated using Satterthwaite’s approximation.

**Table 5:** Intra- and inter-individual predictors of turnover intention.

	b	SE	95% CI	df	t	*p*
Intercept	2.12	0.08	[1.96; 2.28]	46.8	26.33	<0.001
*Level 1 variables _WP_*						
Occasion	−0.05	0.07	[−0.19; 0.09]	78.9	−0.73	0.469
Perceived occupational stress _WP_	0.34	0.15	[0.05; 0.63]	78.1	2.31	0.023
Emotional exhaustion _WP_	0.26	0.10	[0.05; 0.46]	76.1	2.50	0.014
Job satisfaction _WP_	−0.25	0.10	[−0.46; −0.05]	75.2	−2.42	0.018
*Level 2 variables*						
Emotional exhaustion	0.40	0.09	[0.23; 0.57]	53.0	4.72	<0.001
Job satisfaction	−0.32	0.09	[−0.49; −0.15]	48.6	−3.67	<0.001
Peer support	−0.31	0.16	[−0.63; −0.004]	56.3	−2.01	0.049

Note: _WP_ = within-person. Number of observations = 125. Number of participants = 52. Random intercept SD = 0.41. Marginal R^2^ = 0.575. Conditional R^2^ = 0.707. Degrees of freedom were estimated using Satterthwaite’s approximation.

## Data Availability

Data and materials of the current study are available in the OpenScience Framework repository at the following link: https://osf.io/qmd2f/overview?view_only=f63c7ddc22af4c8585b2750c6807c213.

## Supplementary Materials

The following supporting information can be downloaded at https://www.mdpi.com/article/10.3390/healthcare14152316/s1, Table S1: Baseline comparisons between participants retained in the analytical sample and participants lost to follow-up; Table S2: Model including within-person stress variables; Table S3: Model including within-person HSE-MS IT variables; Table S4: Model including within-person motivational variables; Table S5: Interaction analyses testing the buffering hypothesis of the Job Demands–Resources model; Table S6: Robustness analysis of the final multilevel model including demographic and employment-related covariates; Table S7: Alternative Level-2 model specifications; Figure S1: Q–Q plot of the residuals from the final model.

## Abbreviations

The following abbreviations are used in this manuscript:

JD-R	Job Demands–Resources model
POS	Perceived Occupational Stress scale
TI	Turnover Intention
UWES	Utrecht Work Engagement Scale
HSE-MS IT	The Health and Safety Executive Management Standards Indicator Tool
ICC	Intraclass correlation coefficient
