# Supplementary material for: Dynamics of Occupational Well-Being and Turnover Intention in Long-Term Care: A 12-Month Longitudinal Study of Nursing Assistants
Source: Healthcare (Basel). 2026 Jul 31;14(15):2316. doi: 10.3390/healthcare14152316 (PMC13465083; doi:10.3390/healthcare14152316)
Supplement: Supplementary file 1 [file healthcare-14-02316-s001.zip › healthcare-4364099-supplementary.pdf]

Table S1. Baseline comparisons between participants retained in the analytical sample and participants lost to follow-up.

Variable	Retained (N = 52) Mean (SD)/ percentages of groups	Excluded (N = 20) Mean (SD)/ percentages of groups	Test	p	Effect size (Cohen's d/ φ/ Cramér's V/ Rank-biserial correlation)
T0 HSE Demands	2.76 (0.64)	2.79	Student's t	.885	d = 0.04
T0 HSE Managerial Support	3.06 (0.95)	2.67 (0.97)	Student's t	.151	d = -0.41
T0 HSE Peer Support	3.55 (0.78)	3.05 (0.78)	Student's t	.022	d = -0.64
T0 Emotional Exhaustion	3.15 (1.38)	3.74 (1.76)	Mann-Whitney U	.223	r_rb = -0.20
T0 Perceived Occupational Stress	3.08 (1.01)	3.41 (1.01)	Student's t	.224	d = 0.33
T0 Turnover Intention	2.29 (1.35)	2.61 (1.60)	Student's t	.422	d = 0.23
T0 Job Satisfaction	5.11 (1.36)	4.60 (1.57)	Student's t	.188	d = -0.36
T0 Work Engagement	4.88 (1.36)	4.33 (1.08)	Student's t	.133	d = -0.42
Gender	F = 74.5%<br>M = 25.5%	F = 68.8%<br>M = 31.3%	X^2	.650	φ = 0.05
Age	18-29 = 40%<br>30-39 = 16%<br>40-49 = 18%<br>50-59 = 20%<br>≥ 60 = 6%	18-29 = 15.4%<br>30-39 = 38.5%<br>40-49 = 15.4%<br>50-59 = 30.8%<br>≥ 60 = 0%	X^2	.225	V = 0.30
Length of service	0-1 = 28%<br>2-5 = 32%<br>6-10 = 8%<br>10-20 = 24%<br>20-30 = 4%<br>>30 = 4%	0-1 = 33.3%<br>2-5 = 25%<br>6-10 = 25%<br>10-20 = 16.7%<br>20-30 = 0%<br>>30 = 0%	X^2	.561	V = 0.25
Type of contract (long term Yes/No)	No = 23.4%<br>Yes = 76.6%	No = 47.1%<br>Yes = 52.9%	X^2	.067	φ = 0.23

Note: Retained participants were those who completed at least two assessment waves and were therefore included in the longitudinal analyses. Participants who completed only one assessment wave were excluded from the analytical sample. Continuous variables are reported as mean (SD), whereas categorical variables are reported as percentages. Group differences were assessed using independent-samples t-tests, Mann–Whitney U tests (when assumptions for parametric tests were violated), or χ^2 tests, as appropriate. Effect sizes are reported as Cohen's d, rank-biserial correlation, Phi coefficient (φ), or Cramér's V, depending on the statistical test.

Table S2. Model including within-person stress variables.

	b	SE	95% CI	df	t	p
Intercept	2.13	0.14	[1.84; 2.41]	50.9	14.98	<.001
Level 1 variables $_{WP}$						
Occasion	-0.05	0.07	[-0.19; 0.09]	80.7	-0.74	.464
Perceived occupational stress $_{WP}$	0.43	0.14	[0.15; 0.72]	80.6	3.05	.003
Emotional exhaustion $_{WP}$	0.25	0.10	[0.05; 0.45]	78.5	2.50	.014

Note: WP = within-person. Number of observations = 131. Number of participants = 52. Random intercept SD = 0.94. Marginal R^2 = .072. Conditional R^2 = .720. Degrees of freedom were estimated using Satterthwaite's approximation.

Table S3. Model including within-person HSE-MS IT variables.

	b	SE	95% CI	df	t	p
Intercept	2.15	0.16	[1.83; 2.47]	43	13.53	<.001
Level 1 variables $_{WP}$						
Occasion	0.002	0.11	[-0.21; 0.22]	55.6	0.02	.987
Control $_{WP}$	-0.14	0.24	[-0.62; 0.33]	54.7	-0.60	.551
Demands $_{WP}$	-0.44	0.30	[-1.03; 0.15]	54.7	-1.47	.148
Peer Support ^{WP}	-0.02	0.24	[-0.50; 0.47]	54.4	-0.07	.944
Managers' Support ^{WP}	-0.03	0.28	[-0.40; 0.33]	53.9	-0.18	.857
Role ^{WP}	-0.55	0.35	[-1.25; 0.15]	51.6	-1.57	.122
Relationships ^{WP}	-0.30	0.24	[-0.78; 0.19]	55.3	-1.21	.231

Note: WP = within-person. Number of observations = 104. Number of participants = 46. Random intercept SD = 0.94. Marginal R^2 = .051. Conditional R^2 = .645. Degrees of freedom were estimated using Satterthwaite's approximation.

Table S4. Model including within-person motivational variables.

	b	SE	95% CI	df	t	p
Intercept	2.13	0.14	[1.85; 2.41]	49.9	15.06	<.001
Level 1 variables WP						
Occasion	-0.05	0.08	[-0.20; 0.11]	83.3	-0.61	.542
Job Satisfaction WP	-0.48	0.11	[-0.71; -0.26]	82	-4.25	<.001
Work Engagement WP	0.01	0.08	[-0.14; 0.17]	79.9	0.18	.860

Note: WP = within-person. Number of observations = 135. Number of participants = 52. Random intercept SD = 0.92. Marginal R^2 = .054. Conditional R^2 = .660. Degrees of freedom were estimated using Satterthwaite's approximation.

Table S5. Interaction analyses testing the buffering hypothesis of the Job Demands–Resources model.

Interaction term	b	SE	95% CI	t	p
Emotional exhaustion ^{BP} × Peer support ^{BP}	-0.19	0.14	[-0.46; 0.08]	-1.40	.168
Perceived occupational stress ^{WP} × Peer support ^{BP}	0.33	0.29	[-0.23; 0.90]	1.17	.246
Emotional exhaustion ^{WP} × Peer support ^{BP}	0.01	0.20	[-0.40; 0.41]	0.03	.975
Emotional exhaustion ^{BP} × Job Satisfaction ^{BP}	-0.02	0.05	[-0.12; 0.08]	-0.39	.699
Perceived occupational stress ^{WP} × Job Satisfaction ^{BP}	-0.10	0.15	[-0.40; 0.20]	-0.67	.503
Emotional exhaustion ^{WP} × Job Satisfaction ^{BP}	-0.17	0.09	[-0.34; 0.005]	-1.92	.058

Note: Each interaction term was tested in a separate linear mixed-effects model including the same fixed effects and random intercept structure as the primary model. Models were estimated separately to avoid overparameterization given the sample size.

Table S6. Robustness analysis of the final multilevel model including demographic and employment-related covariates.

	Fixed effect (*b*)	SE	95% CI	t	p
Intercept	2.25	0.15	[1.94; 2.55]	14.47	<.001
Level 1 variables $_{WP}$					
Occasion	-0.05	0.07	[-0.20; 0.10]	-0.67	.507
Perceived occupational stress $_{WP}$	0.35	0.15	[0.05; 0.65]	2.29	.025
Emotional exhaustion $_{WP}$	0.26	0.10	[0.05; 0.47]	2.47	.016
Job Satisfaction $_{WP}$	-0.24	0.11	[-0.46; -0.03]	-2.26	.027
Level 2 variables					
Emotional exhaustion	0.38	0.10	[0.18; 0.57]	3.82	<.001
Job Satisfaction	-0.31	0.10	[-0.50; -0.11]	-3.16	.003
Peer support	-0.30	0.18	[-0.67; 0.06]	-1.65	.106
Gender 1-0	-0.03	0.24	[-0.50; 0.43]	-0.14	.887
Age (30–39 vs 18–29)	0.06	0.28	[-0.49; 0.61]	0.22	.827
Age (40–49 vs 18–29)	-0.03	0.26	[-0.54; 0.49]	-0.11	.911
Age (50–59 vs 18–29)	0.13	0.25	[-0.36; 0.62]	0.53	.601
Age (≥60 vs 18–29)	-0.10	0.43	[-0.95; 0.76]	-0.22	.824
Contract (permanent vs fixed term)	-0.53	0.22	[-0.96; -0.10]	-2.44	.019

Note: WP = within-person. Random intercept SD = 0.41. Marginal R^2 = .588. Conditional R^2 = .712.

Table S7. Alternative Level-2 model specifications.

	**Main**	**POS**	**POS+UWES**	**POS+UWES+ Manager Support**	**UWES**	**Manager Support**
Predictor						
bp Burnout	0.40***	—	—	—	0.49***	0.43***
bp POS	—	0.27*	0.43***	0.46***	—	—
bp Job Satisfaction	-0.32***	-0.42***	—	—	—	-0.34***
bp UWES	—	—	-0.26**	-0.27*	-0.16	—
bp Peer support	-0.31*	-0.43*	-0.41*	—	-0.32	—
bp Manager support	—	—	—	-0.26	—	-0.16
Fit						
Marginal R^2	.575	.493	.423	.423	.520	.584
ICC	.312	.426	.460	.530	.365	.371
AIC	305	319	324	324	311	306
BIC	333	347	352	352	340	334

Note: p < .05 *, p < .01 **, p < .001 ***. Only the between-person (Level-2) predictors that differed across model specifications are shown. All models included the same within-person predictors (occasion, perceived occupational stress, emotional exhaustion, and job satisfaction) and a random intercept for participants. Estimates for the within-person predictors remained highly comparable across all alternative specifications and are therefore not reported.

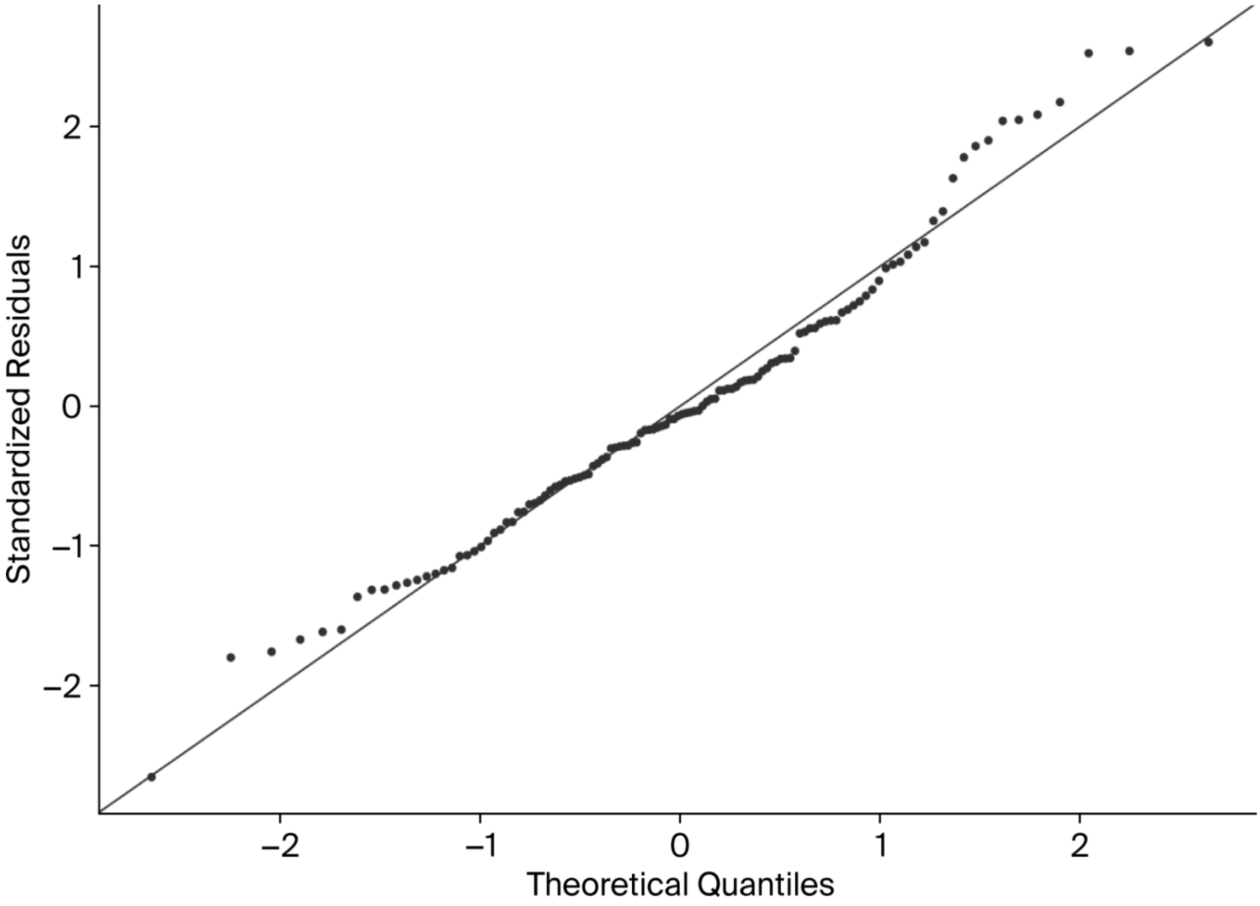

Figure S1. Q–Q plot of the residuals from the final model.
